# Supplementary figures and images for: Multi-Time Point Transcriptome Analysis and Functional Validation Revealed Bol4CL41 Negatively Regulates Black Rot Resistance in Cabbage
Source: Int J Mol Sci. 2025 Jun 26;26(13):6179. doi: 10.3390/ijms26136179 (PMC12249714; doi:10.3390/ijms26136179)

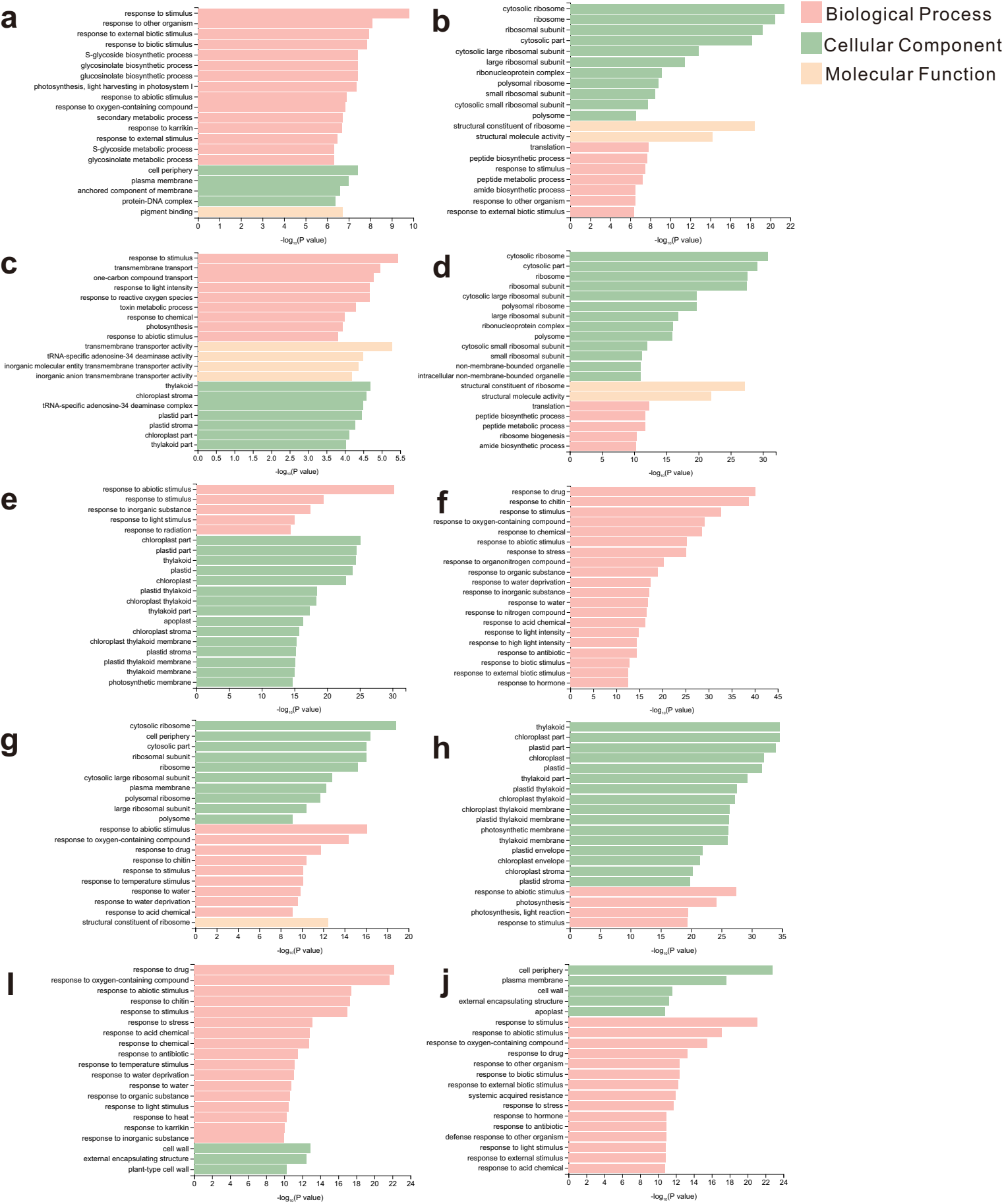

Supplement: Supplementary file 1 [file ijms-26-06179-s001.zip › Figure_S1.pdf]

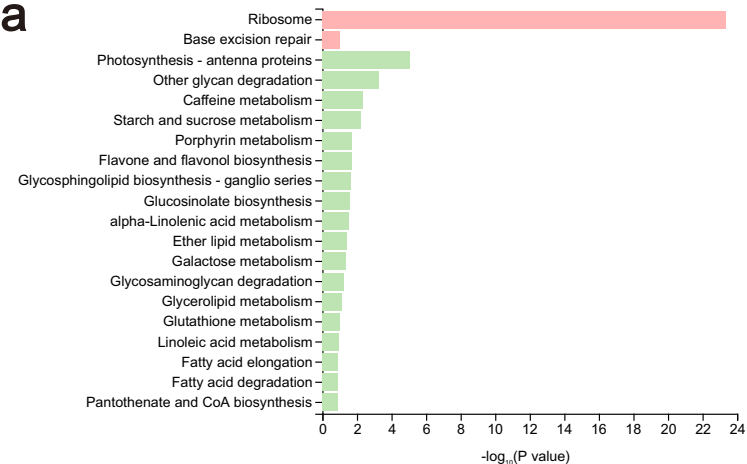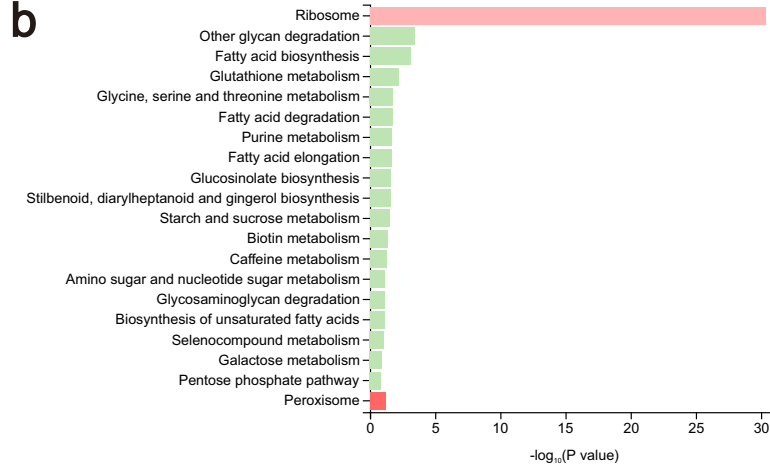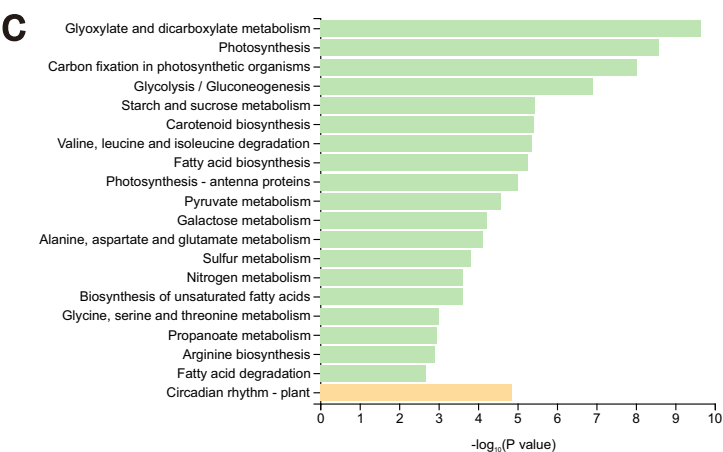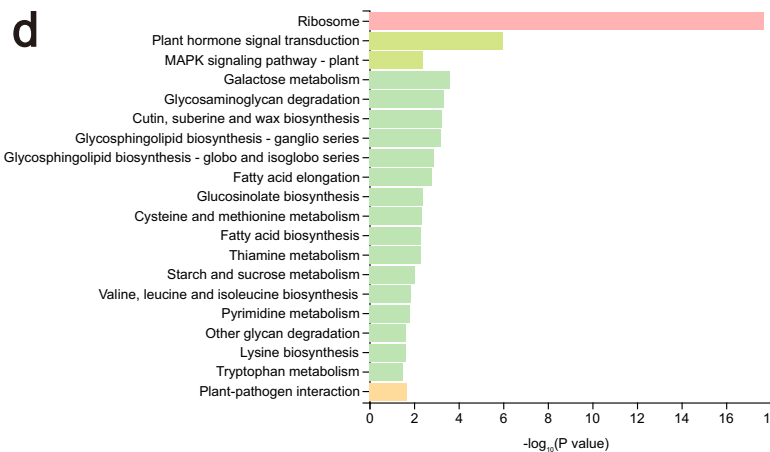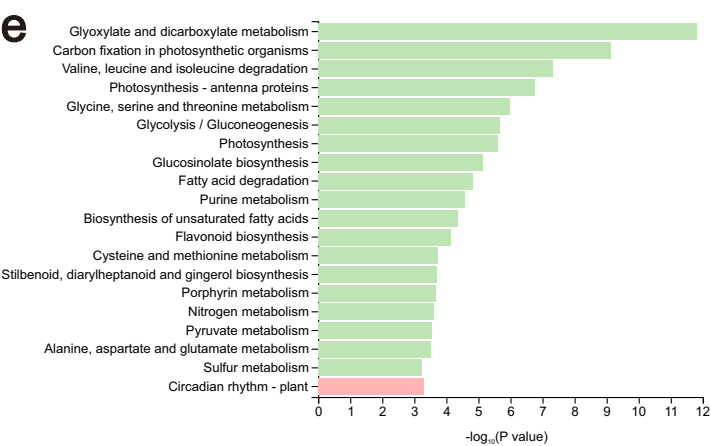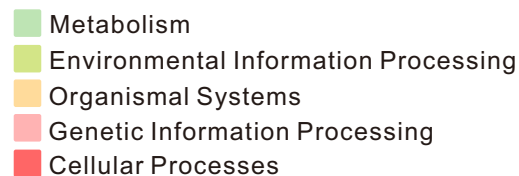

Supplement: Supplementary file 1 [file ijms-26-06179-s001.zip › Figure_S2.pdf]
